# Five Days Periodic Fasting Elevates Levels of Longevity Related *Christensenella* and Sirtuin Expression in Humans

**DOI:** 10.3390/ijms22052331

**Published:** 2021-02-26

**Authors:** Stephanie Lilja, Carina Stoll, Ulrike Krammer, Berit Hippe, Kalina Duszka, Tewodros Debebe, Ingrid Höfinger, Jürgen König, Angelika Pointner, Alexander Haslberger

**Affiliations:** 1 Department of Nutritional Sciences, University of Vienna, 1010 Wien, Austria; stephanie.lilja@univie.ac.at (S.L.); carina.stoll@gmx.at (C.S.); U.krammer92@gmx.at (U.K.); berit.hippe@univie.ac.at (B.H.); kalina.duszka@univie.ac.at (K.D.); juergen.koenig@univie.ac.at (J.K.); angelika.pointner@univie.ac.at (A.P.); 2 Biomes NGS GmbH, 15745 Wildau, Germany; Tewodros-debebe.aklilu@biomes.world; 3 Monastery Pernegg, 3753 Pernegg, Austria; ingrid.hoefinger@gmx.at

**Keywords:** Buchinger fasting, gut microbiota, sirtuin

## Abstract

Periodic fasting (PF) is an increasingly popular approach that assists in the management of metabolic and inflammatory diseases as well as in preventing mechanisms involved in aging. However, little is known about the effects of fasting on gut microbiota and its impact on the epigenetic regulation of metabolically relevant enzymes, especially sirtuins (SIRTs). We analyzed the effect of periodic fasting on the human gut microbiota, SIRTs expression, and mitochondrial content in 51 males and females. The participants fasted under supervision for five consecutive days following the Buchinger fasting guidelines. Ketogenesis, selected mRNAs, miRNAs, mitochondrial (mt) DNA, and gut composition were analyzed before and after PF. PF triggered a significant switch in metabolism, as indicated by the increase in ß-hydroxybutyrate (BHB) and pyruvate dehydrogenase kinase isoform 4 (*PDK4)* expression in the capillary blood. MtDNA, *SIRT1*, *SIRT3*, and *miRlet7b-5p* expression in blood cells were elevated, whereas *SIRT6* and *miR125b-5p* were not affected. Following fasting, gut microbiota diversity increased, and a statistically significant correlation between *SIRT1* gene expression and the abundance of *Prevotella* and *Lactobacillus* was detected. The abundance of longevity related *Christensenella* species increased after fasting and inversely correlated with age as well as body mass index (BMI). Thus, this represents the first study that showing that fasting not only changes the composition of the gut microbiota, making it more diverse, but also affects SIRT expression in humans.

## 1. Introduction

The occurrence of metabolic disorders has dramatically increased during recent decades in developed and developing countries. Obesity, type 2 diabetes, hypertension, cardiovascular disease, gastrointestinal disorders, and cancer often correspond to aging and contribute to increased mortality [1,2]. Restrictive diets (RD), including caloric restriction (CR), time-restricted feeding, and intermittent fasting, are known to retard age-related diseases, thus, increasing life span, at least in animal models [3]. Whether RDs extend lifespan in humans is unknown. In general, a reduction in caloric intake reduces metabolic rate and oxidative stress, improves insulin sensitivity, and alters neuroendocrine and sympathetic nervous system functioning; all known to be altered in obesity and during the progression of aging [4,5]. In contrast to RD which usually lasts long-term, periodic fasting (PF)—such as Buchinger fasting—involves a daily energy intake of a maximum of 250 kcal for approximately one week. Although, under medical supervision, it can last up to three weeks [6,7].

During fasting, glucose and glycogen stores are depleted, followed by enhanced lipolysis to supply the peripheral tissue with free fatty acids, which later can be converted into ketone bodies, mainly in the mitochondria of the liver [8]. One of the ketone bodies, ß-hydroxybutyrate (BHB), controls cellular signaling, regulates gene expression, is also post-transcriptionally active, and minimizes neurological impairments associated with aging [9]. Concentrations of BHB in plasma can rise from 0.6 mM during RD to up to 1 mM after 20 h of nutrition depletion and up to 7 mM following one week of PF [10]. 

The beneficial effects of fasting occur by modulating the activity of multiple pathways, including nutrient responsive pathways, such as the insulin/insulin-like growth factor (IGF-1) pathway, adenosine monophosphate-activated protein kinase (AMPK), as well as different classes of histone deacetylases (HDACs), and suppression of the nucleotide-binding oligomerization domain (NOD)-like receptor (NLR), pyrin domain-containing protein 3 (NLRP3) inflammasome [9]. Decreased insulin signaling activates AMPK and mammalian Forkhead-Box-O (FoxO) proteins, such as FoxO1 and FoxO3, which stimulate the expression of many genes involved in autophagy, including sirtuins (SIRTs), the mammalian homolog of the silent mating type information regulation (SIR) genes present in lower eukaryotes [11]. AMPK promotes an intracellular increase in nicotinamide adenine dinucleotide (NAD^+^) levels, the rate-limiting substrate for SIR2, which is one of the critical mediators of CR-induced lifespan extension in yeast. In mammals, seven types of SIRTs have been identified (SIRT1–7) [11,12], which can be found in different parts of the cells, and have multiple functions including DNA repair, cell survival, metabolism, lipid and glucose homeostasis, stress resistance, as well as insulin secretion, mostly via their HDACs activities. SIRT1 is the most intensively studied member of the SIRT family and it is associated with longevity in animal models. AMPK and SIRT1 both regulate each other’s activities and share many common targets and functions [13,14]. Both AMPK and SIRT promote mitochondrial biogenesis and functioning. Consequently, they increase cells ability to generate ATP, diminish oxidative stress, and other potentially adverse cellular events [11,12,13,14].

SIRTs are also expressed in the gastrointestinal tract. Mice lacking *SIRT3* expression have altered gut microbiomes, exhibited a higher inflammatory level, and increased intestinal epithelial damage [15]. A decline in SIRT expression and decreased intestinal microbial diversity is associated with aging [16]. Dysbiosis or a less diverse gut microbiome is associated with aberrations of gut barrier integrity and inflammation, which contribute to pathogenesis like type 2 diabetes, fatty liver, and hepatic steatosis, atherosclerosis, cardiovascular disease, which are comorbidities for obesity and aging [17]. The intestinal microbiome impacts gene expression inducing epigenetic changes and regulates activity of G-protein coupled receptors via short-chain fatty acids (SCFAs). SCFAs are gut microbiota metabolites generated by the fermentation of dietary fiber. One of these SCFAs—butyrate—inhibits class I HDACs, epigenetically induces the proliferation and differentiation of immune cells and upregulates the activity of the adiponectin-mediated AMPK pathway that stimulates mitochondria biogenesis and fatty acid oxidation [1].

However, RD and PF can affect gene expression through multiple mechanisms, such as chromatin modification, mRNA transcription, and mRNA translation. Control of the expression of regulatory RNAs, such as microRNAs (miRNAs), is an important determinant in this regard [18].

Health-span results from the interaction of multiple factors including genetic and epigenetic, as well as microbiota [19]. Based on previous reports showing that RDs have a beneficial impact on human health, which was exemplified by clinical parameter results and an enhanced health-span [20], we conducted a study to investigate the potential impact of Buchinger fasting on age-related pathways and the microbiome. To our knowledge, so far, most scientific studies have focused on RD or intermittent fasting and only a few have investigated the effects of PF in consideration of the human intestinal microbiota; however, none of these have done so by focusing on longevity related genes.

## 2. Results

### 2.1. Fasting Results in Ketogenesis, Weight loss, and Increased Levels of mtDNA

**Figure 1:**
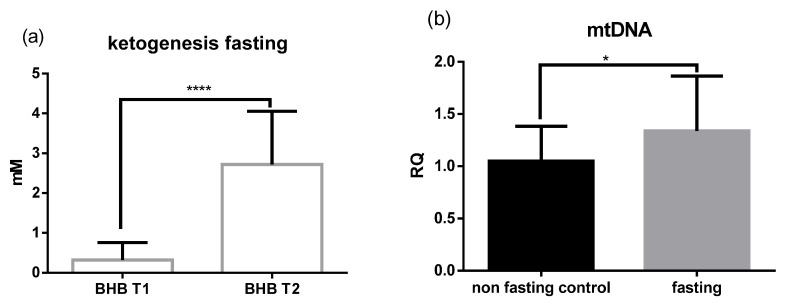
Periodic fasting (PF) increases ketogenesis and mitochondrial (mt)DNA. (**a**) ß-hydroxybutyrate (BHB) levels were measured in the blood of fasting subjects before (T1) and after (T2) the intervention (*p* < 0.01). (**b**) Relative quantification (RQ) of mtDNA was measured in blood. The results are expressed as mean +/− SD. Statistical significance was determined using paired *t*-test for parametric values and Wilcoxon test for nonparametric values.

Male (*n* = 5) and female (*n* = 15) study participants underwent five days of PF following the Buchinger fasting protocol, while other groups of male (*n* = 11) and female (*n* = 20) subjects served as non-fasting controls. Approximately half of the total study population (52,9%) had a body mass index (BMI) lower than 25 kg/m^2^, 35.3% were defined as overweight and 11.8% were obese (Table 1). After PF, blood BHB significantly increased from 0.2 to 5.7 mM (*p* < 0.01) (Figure 1a). Due to the intervention, a mean weight loss of 4.26 kg was recorded (Table 1). Relative mitochondrial (mt) DNA content in the blood was significantly higher in the fasting group compared to the non-fasting control group (*p* < 0.05) (Figure 1b).

### 2.2. PF Affects mRNA and miRNA Expression

**Figure 2:**
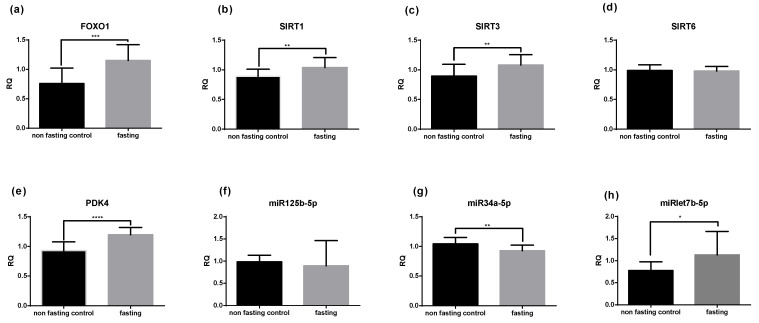
PF affects gene expression in blood cells. Relative quantification (RQ) after the intervention compared to the non-fasting control group of selected mRNA (*FoxO1*, *SIRT1*, *SIRT3*, *SIRT6* and *PDK4* (**a**–**e**)) and miRNA (*miR125b-5p*, *miR34a-5p* and *miRlet7b-5p* (**f**–**h**)). The results are expressed as mean +/− SD. Statistical significance was determined using paired *t*-test for parametric values and Wilcoxon test for nonparametric values.

Selected mRNA and miRNA levels were assessed in the capillary blood of the study subjects. After PF, changes in gene expression were detected for all of the selected genes, apart from *SIRT6*, and *miR125b-5p.* The levels of *FoxO1*, *SIRT1*, *SIRT3*, and *miRlet7b-5p* were significantly increased compared to the non-fasting controls, whereas *miR34a-5p* levels were reduced (*p* < 0.01) (Figure 2a–h).

**Figure 3:**
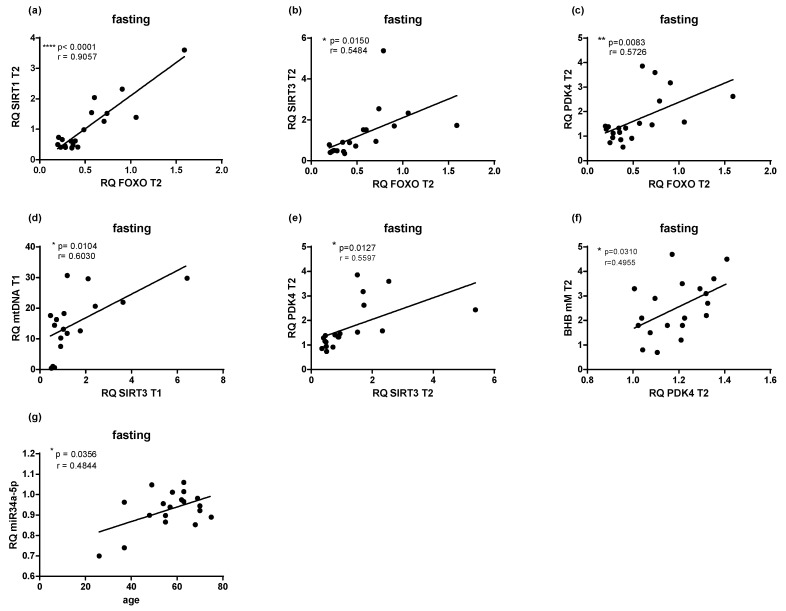
PF results in correlations between gene expression, age, ß-hydroxybutyrate (BHB), and mitochondrial (mt)DNA. Scatterplots illustrate Pearson correlation of different gene expressions with other markers for the fasting population and different timepoints (**a**–**g**). Statistical significance was defined as a *p*-value below 0.05.

PF resulted in significant correlations between the expression of *FoxO1* with *SIRT1*, *SIRT3*, and *PDK4* (Figure 3a–c). Similarly, *SIRT3* expression positively correlated with mtDNA levels, but only at the baseline (*p* < 0.05) (Figure 3d). Moreover, PDK4 positively correlated with BHB blood concentrations and SIRT3 expression after PF (*p* < 0.02) (Figure 3e,f). Although attenuating *miR34a-5p* expression would indicate higher *SIRT1* levels, no association was obtained, yet increasing age leads to overexpression of miR34a-5p (*p* < 0.05) (Figure 3g).

### 2.3. Gut Microbiota Composition Changes and Diversity

**Figure 4:**
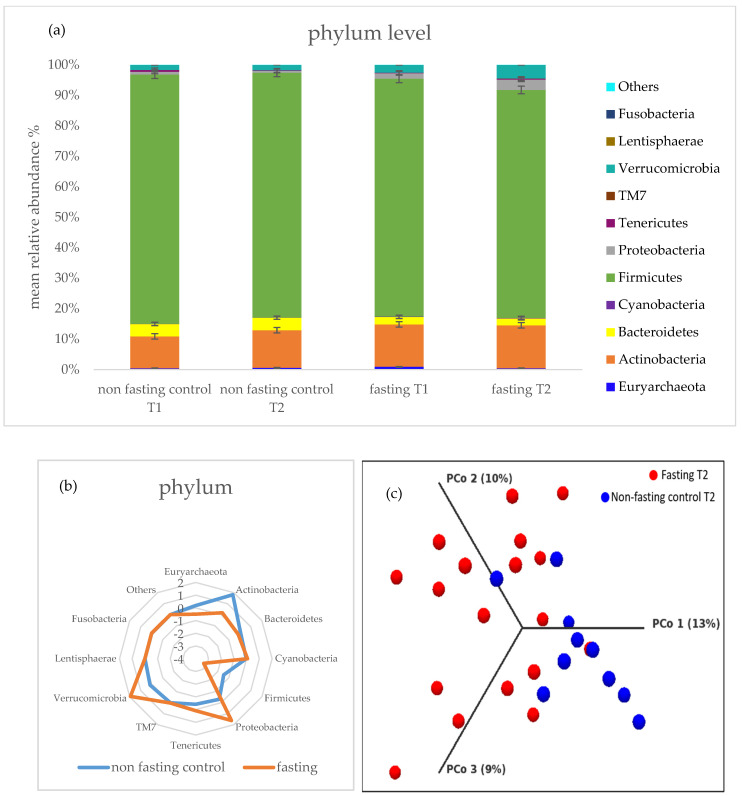
The dissimilarity of the microbiota composition of the non-fasting control and fasting group. (**a**) Bar charts of sequencing data given in mean +/− SD relative bacteria abundance in % at phylum level for non-fasting and fasting group. (**b**) Major differences between non-fasting and fasting groups at the phylum level. Values are given as the mean abundance of T2–T1. (**c**) PCoA based on Bray–Curtis dissimilarity index showing cluster for fasting and non-fasting group at T2. Permutational multivariate analysis of variance (PERMANOVA; *p* = 0.00004) was applied for the analysis.

Gut composition and metabolomics were assessed in stool samples. PF resulted in microbiota composition changes. At the phylum level *Tenericutes*, *Verrucomicrobia*, *Cyanobacteria*, *Proteobacteria*, *TM7*, and *Fusobacteria* were affected; however, only the last three were statistically significant (*p* < 0.05) (Figure 4a). A strong statistical trend was observed for the change in abundance of *Euryarchaeota* and *Cyanobacteria* before and after PF. The level of the latter was increased after the intervention, whereas the level of *Euryarchaeota* was reduced after PF (Figure 4a). No significant changes were seen for *Actinobacteria*, *Bacteroidetes*, and *Firmicutes* (Figure 4a). The non-fasting group showed no changes in microbiota composition of any of the phyla (Figure 4a). Comparing the fasting and non-fasting group, *Verrcoumicrobia*, *Firmicutes*, *Actinobacteria*, and *Proteobacteria* are the phyla with the strongest differences at T2 (Figure 4b). 

The Shannon diversity index was used to calculate α-diversity, but sequencing results showed no significant differences between non-fasting and fasting groups and within the different timepoints. The dataset was further subjected to principal coordinates analysis (PCoA). Although overlapping, PCoA showed a significant grouping of band patterns according to the two groups of study participants at T2 (Figure 4c). 

**Figure 5:**
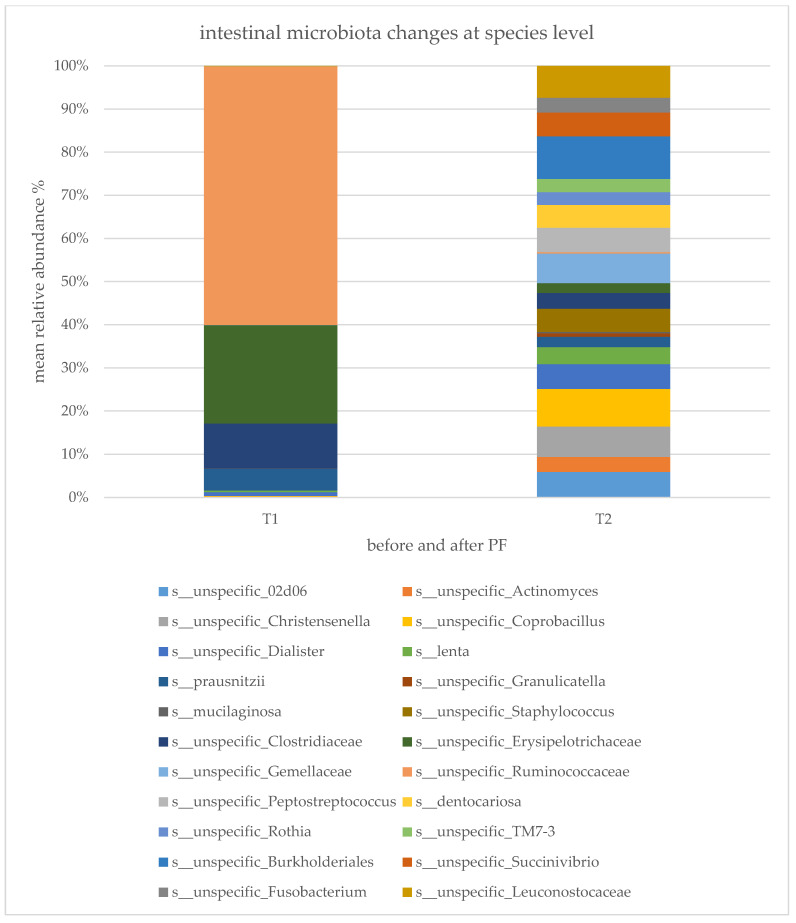
Microbial changes at species level before and after PF. Bar charts of all statistically significant changes of the sequencing data at species level given in mean relative bacteria abundance in % for the fasting group. Statistical significance was determined using paired *t*-test for parametric values and Wilcoxon test for nonparametric values and defined as *p* < 0.05.

All statistically significant changes at the species level for the fasting versus control groups are illustrated in Figure 5, which demonstrates strong differences in the microbiota composition at the species level before and after the fasting intervention. Significant decreases were observed for: s__unspecific_02d06; s__unspecific_Dialister; s__prausnitzii; s__unspecific_Clostridiaceae; s__unspecific_Ruminococcaceae. Elevations were observed for: s__unspecific_Actinomyces; s__unspecific_Christensenella; s__unspecific_Coprobacillus; s__lenta; s__unspecific_Granulicatella; s__mucilaginosa; s__unspecific_Staphylococcus; s__unspecific_Erysipelotrichaceae; s__unspecific_Gemellaceae; s__unspecific_Peptostreptococcus; s__dentocariosa; s__unspecific_Rothia; s__unspecific_TM7-3; s__unspecific_Burkholderiales; s__unspecific_Succinivibrio; s__unspecific_Fusobacterium; s__unspecific_Leuconostocaceae (Figure 5).

### 2.4. PF Decreases Firmicutes/Bacteroidetes Ratio and Addresses Microbial Metabolites

**Figure 6:**
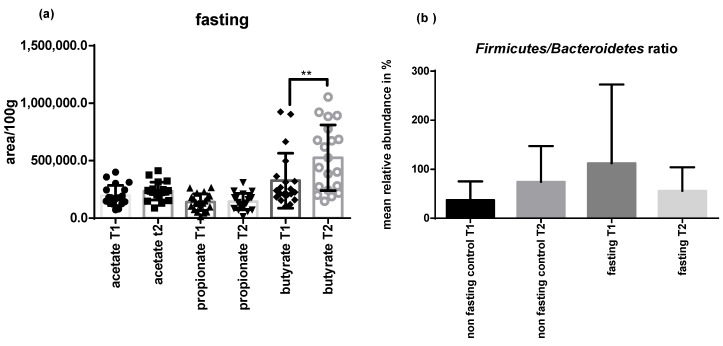
PF increased levels of butyrate and changed *Firmicutes/Bacteroidetes* ratio. Short-chain fatty acids (SCFAs) amount comparison of fasting group T1 versus T2 (**a**) Firmicutes/Bacteroidetes ratio increased for the non-fasting group and decreased after PF, although not statistically significant (**b**). The results are expressed as mean +/− SD. Statistical significance was determined using paired *t*-test for parametric values and Wilcoxon test for nonparametric values.

At the end of the intervention, a positive correlation was observed for abundance in *Tenericutes* and the amount of butyrate produced, which significantly increased after the fasting period (*p* < 0.05) (Figure 6a).

The *Firmicutes/Bacteroidetes* ratio decreased in the fasting and elevated in the non-fasting group (Figure 6b) but no correlations were seen with BMI or weight.

### 2.5. Microbial Changes at Genus Level and Correlations

**Figure 7:**
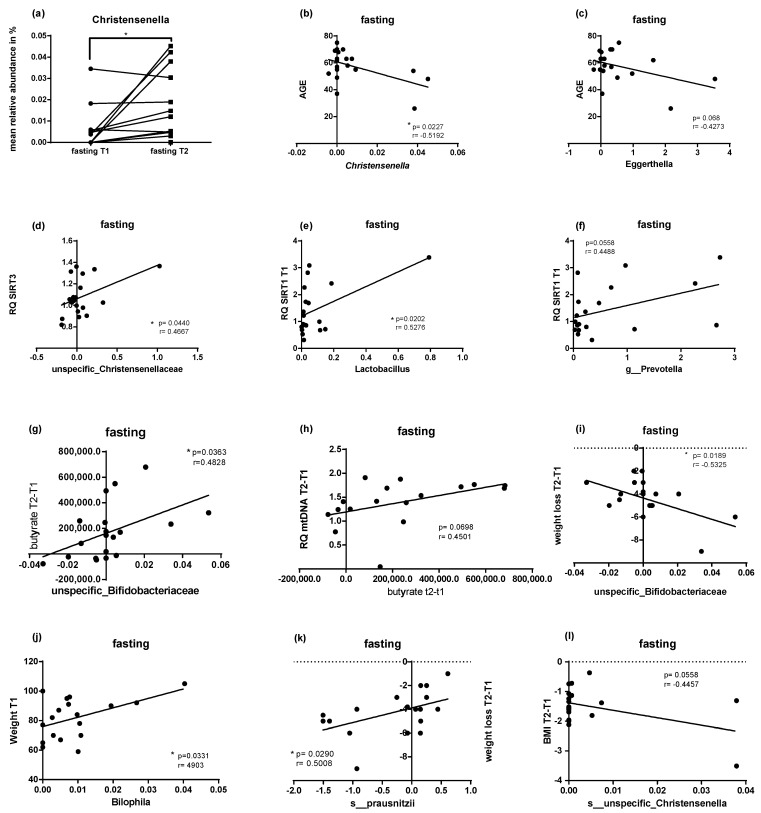
Correlation of gut microbiota at genus level with other biomarkers. *Christensenella* amount increased after PF described as fasting T1 and fasting T2. Statistical significance was determined using paired *t*-test for parametric values (**a**) Several Pearson correlations of gut microbiota with age, weight, weight loss, gene expression, butyrate, and BMI at different timepoints are illustrated from (**b**–**l)**. Statistical significance was defined as a *p*-value below 0.05.

At the genus level, PF triggered changes in the abundance of *Actinomyces*, *Granulicatella*, *Roseburia*, *Rothia*, *Rominococcus*, *Eggerthella*, and *Christensenella* (*p* < 0.05). The levels of longevity related *Christensenella* increased after PF (Figure 7a). Age negatively correlated with the abundance for *Christensenella* (Figure 7b), *Eggerthella* showed a similar trend; however, this was not statistically significant (*p* = 0.068) (Figure 7c). The abundance of *Christensenellaceae* positively correlated with *SIRT3* expression (*p* < 0.05) (Figure 7d). Participants with a higher abundance of *Prevotella* or *Lactobacillus* had higher levels of *SIRT1* expression with a statistically significant correlation (*p* = 0.020) for *Lactobacillus* and a strong trend (*p* = 0.058) for *Prevotella* (Figure 7e,f). At the genus level, unspecific *Firmicutes* and *Bifidobacteriaceae* correlated with the PF-triggered increase in the levels of butyrate (*p* < 0.05) (Figure 7g). The higher amount of butyrate showed a trend for correlation with higher mtDNA (*p* = 0.0698) (Figure 7h). *Bifidobacteriaceae* correlated with higher levels of weight loss for the study population with increased abundance of this genera (*p* = 0.0189) (Figure 7i). *Rothia* showed a similar result but only as a trend (*p* = 0.07). At the baseline, *Bilophila* was more abundant in participants with higher weight for the fasting group (*p* = 0.0331) (Figure 7j). Interestingly, at the species level, the amount of *Faecalibacterium prausnitzii* correlated with a lower level of weight loss (Figure 7k). Nevertheless, unspecific *Christensenella*, the amount of which was significantly ameliorated after periodic fasting, showed a strong correlation with a lower BMI (*p* = 0.0558) (Figure 7l).

## 3. Discussion

Restrictive dietary protocols have proven to elicit beneficial effects on health. PF reduces blood pressure, insulin resistance and improves wellbeing [7,20,21]. Only a few studies have investigated PF in humans and focused on clinical blood parameters, microbiota composition changes, or longevity-related parameters.

As expected, our results show that nutrition depletion leads to weight loss and a switch in metabolism, as documented by the increased *PDK4* expression. Significant differences between the fasting and control group were seen for *SIRT1*, *SIRT3*, *FoxO1*, *PDK4*, *miR34a-5p* and *miRlet7b-5p*, *BHB* and *mtDNA*. However, no significant differences were seen between gender or BMI groups, in the fasting intervention, possibly caused by too few a number of participants or different characteristics of the control group.

In addition, ketone body production was increased during PF which is characteristic of prolonged fasting. BHBs act on hydroxy-carboxyl acid receptors (HCA), in particular, on HCA2/GPR109a, which is a G-protein-coupled receptor. GPR109a has the capacity to prevent metabolic and inflammatory diseases, including type 2 diabetes, by suppressing the inflammasomes [22,23,24]. Therefore, GPR109a is a factor that represents increased health and lifespan, with it showing potential to be used as a therapeutic target for the prevention of type 2 diabetes, obesity, and inflammation [25].

Similar to the study conducted by Cannataro et al. [24] we observed an increase in *miRlet7b-5p* levels after PF. An overexpression of *miRlet7b-5p* leads to lower levels of triglycerides and insulin secretion by targeting the retinoid x receptor (RXR) and insulin receptor substrate, respectively. Further, *miRlet7b-5p* is involved in multiple metabolic regulatory processes, such as adipogenesis and fat metabolism [24], whereas an increase in *miR34a-5p* can lead to mitochondrial dysfunction [26]. Moreover, *miR34a-5p* acts as a pro-senescence factor by leading to increased inflammation and reduced mitochondrial content [26,27,28]. Overexpression of *miR34a* is known to correlate with aging and reduced levels of *SIRT1* [29]. The former was confirmed by our study. After five days of PF, we observed decreased expression of *miR34a-5p*; however, a significant correlation with its main target—*SIRT1*—was not detected, possibly because the study population was too small.

Sirtuins, especially *SIRT3*—located in the mitochondria—regulate many pathways involved in fasting metabolism, e.g., ketone body production, via deacetylating the enzyme 3-hydroxy-3-methylglutaryl-CoA synthase; the rate-limiting step in β-hydroxybutyrate synthesis [30,31]. This is reflected in our results, where non-fasting controls had significantly lower *SIRT3* expression compared to fasting subjects. In addition, positive correlations between levels of BHB and *PDK4* and the latter with SIRT3 were observed in our study. Increased *PDK4* gene expression is usually observed during fasting, which leads to inhibition of the pyruvate dehydrogenase complex, conversion of pyruvate to acetyl CoA decreases, and at the same time ß-oxidation increases to provide energy [32]. Shimazu et al. [31] reported that mice lacking *SIRT3* showed decreased BHB levels during fasting. Additionally, *SIRT3* contributes to mitochondrial quality and biogenesis, by activating antioxidative factors, such as *FoxO* and superoxide dismutase [33]. Correspondingly, we found positive correlations between *FoxO1* and *SIRT1*, *SIRT3* and *PDK4* in the intervention group. Likely due to its involvement in mitochondrial biogenesis, *SIRT3* expression correlated with mtDNA content in the blood.

The levels of *SIRT6*, also involved in aging and metabolism, did not alter during the fasting intervention, and no differences were seen between the PF and control groups. Nevertheless, *SIRT1* gene expression increased after PF and significant changes were seen between the subject groups. Overexpression of this gene is associated with attenuated adipogenesis and increased lipolysis. *SIRT1* can also be expressed in the gut, where it exerts anti-inflammatory effects in acute intestinal inflammation [1]. Harada et al. [34] identified the strain *Lactobacillus brevis* T2102 as an *SIRT1* activator in the gut. Consistent with this finding, we found a positive correlation of participants with a higher amount of *Lactobacillus* and *Prevotella* and *SIRT1* expression at baseline. This correlation was not observed after the intervention, thus, a major switch in the gut microbiome occurs after nutrition depletion. Furthermore, abundance changes were seen for *Proteobacteria*, *TM7*, and *Fusobacteria*. Although overlapping, two different clusters were seen for the intervention groups at the phylum level. The *Firmicutes* to *Bacteroidetes* ratio decreased after PF, but contrary to the study by Mesnage et al. [7], in our outcomes, *Firmicutes* remained as the dominant phylum. At the species level, consistent with the study by Wilhelmi de Toledo et al. [21] but not with that by Remely et al. [20], we saw a significant decrease in the level of *Faecalibaterium prausnitzii* after PF. This important butyrate producer is usually associated with a lower BMI and body weight, whereas we found the opposite effect for the fasting group [35]. The family *Bifidobacteriaceae*, another important butyrate producer, which declines with aging, is associated with lower BMI and higher weight loss [36], which is consistent with our results. Another highly interesting finding in our study considers *Christensenella*, which has recently been referred to as a longevity-relevant gut microbiota as it is more frequently found in centenarians [37]. The abundance of *Christensenella* is usually influenced by age, diet, and genetics [37]. In our study population, the abundance of this species decreased with age and higher abundances were inversely correlated with BMI, similar to previous reports [37,38]. Nevertheless, after PF, a significant increase was observed for *Christensenella.* As a butyrate producer, *Christensenella* might have contributed to the significant generation of butyrate following PF. Correspondingly to the beneficial impact of butyrate on mitochondrial function [39,40], we observed a positive trend between stool butyrate and mtDNA content in the blood of the intervention group.

Aging and obesity are associated with lower bacterial diversities and altered metabolic pathways, which are involved in nutrient harvesting and energy production [20]. After PF, we observed a more diverse composition of gut microbiota at the species level, which was distinctive from the composition of the control group. Together, these findings indicate that PF increases the expression of genes and the diversity of gut microbiota relevant for longevity.

Results from larger fasting and control groups are desirable, however, such type of study is difficult to coordinate at the same time and in a single given location. Sustainable effects after fasting are of high interest and promise to be useful for further therapeutic approaches, thus, we are already investigating this topic.

## 4. Material and method

### 4.1. Experimental Design and Dietary Intervention

A total of 55 participants were enrolled for a five-day fasting study performed at the University of Vienna, Department of Nutritional Sciences, in cooperation with the Monastery of Pernegg (Austria). All study participants gave written consent for the use of data. The study population comprised participants, who decided to participate in the fasting group (*N* = 24) and a non-fasting control group (*N* = 31). Dropouts were only present in the fasting intervention, due to a lower tolerability of nutrition and this consequently reduced the group to a total number of 20 participants. During fasting, the subjects were supervised by a fasting coach in the Monastery in Pernegg. Following Buchinger fasting, all subjects were asked to drink 2–3 L of water or non-energy herbal teas daily. Furthermore, an organic freshly squeezed fruit juice (250 mL) was served at noon and a vegetable soup (liquid only) in the evening. To remove intestinal remnants of the last meals, the intestinal tract was emptied through the intake of a laxative supervised by the fasting coach. The study population was between 23 and 75 years (mean 45.24). The mean BMI was 25.93 ± 3.93 kg/m^2^ and the mean weight was 75.9 ± 12.85 kg. Only four participants indicated to be smokers. Physical activity and stress load including age and gender were determined not to be confounding factors. In total, 16 males and 35 females participated in the study population. Participants supplementing probiotics, antibiotics, or sirtuin-activating compounds/medicine such as metformin were excluded from the study.

### 4.2. Sample Collection

A food frequency questionnaire, general health questionnaire, dried blood spots, and stool samples were collected from the total population. Blood ketone body levels were measured before the beginning of fasting and before the fast break—for the fasting group only. The stool samples were collected before starting the fasting and from the first stool after the fasting break. After collection, stool samples were immediately stored at −80°C. In accordance with the declaration of the Viennese Human Ethics Committee, all study participants gave written consent for the use of data generated during the study. The beginning of the intervention is defined as time point 1 (T1). For fasting, the end of the intervention was defined as time point 2 (T2).

### 4.3. BHB Measurement

GK Dual Blood glucose and ketone meters (Swiss Point of Care, Zurich, Switzerland) were used to measure BHB, from the fasting group only, using blood drops from the finger for both timepoints.

### 4.4. DNA and RNA Extraction

Capillary blood drops were collected on Whatman^®^ protein saver cards (Sigma Aldrich, St. Louis, MO, USA), and are described as dried blood spots (DBS). Total DNA and RNA were isolated from DBS using MagMAX FFPE DNA/RNA ultra-kit via a KingFisher Duo Prime purification system (both Thermo Fisher Scientific, Waltham, MA, USA). A nanodrop ND-1000 spectrophotometer (Nanodrop, Wilmington, DE, USA) was used for quantity and quality checks of DNA and RNA samples. 

### 4.5. Mitochondrial DNA

Relative mitochondrial DNA content was determined in genomic DNA isolated from the DBS. For qPCR, a StepOne Plus real-time PCR Detection System (Applied Biosystems, Waltham, MA, USA) and single-copy gene primers, mtDNA primers (Biomers, Ulm, Germany), and a LightCycler^®^ 480 Sybr^®^Green I master mix (Roche, Penzberg, Germany) were used.

Relative mtDNA content was calculated using the formula 2^−ΔΔCt^ (ΔCt = Ct^mtDNA^-Ct^singlecopygene^; ΔΔCt = ΔCt^T2^-ΔCt^T1^) as described elsewhere [41,42]. The sequences of the forward and reverse mitochondrial primers were: CAT CTG GTT CCT ACT TCA GGG and TGA GTG GTT AAT AGG GTG ATA GA. Following primers sequence were used for forward and reverse single-copy gene: CAG CAA GTG GGA AGG TGT AAT CC and CCC ATT CTA TCA TCA ACG GGT ACAA. An initial heating step of 95 °C for 10 min was followed by 40 cycles of 95 °C for 15 s and 60 °C for 1 min.

### 4.6. mRNA and miRNA Expression

Genes of relevance to RD and longevity were selected. Changes in mRNA expression were determined using available primers (Thermofisher, Waltham, MA, USA). LunaScript RT SuperMix Kit (New England BioLabs, Frankfurt, Germany) and TagMan Advanced miRNA cDNA synthesis kit (Thermofisher, Waltham, MA, USA) was used for cDNA synthesis of mRNA and miRNA. cDNA synthesis was performed using MultiGene gradient Thermal Cycler (Labconsulting, Vienna, Austria). A total of 10 μL reactions were run in duplicates using TaqMan Fast advanced Mastermix (Thermofisher, Waltham, MA, USA) and StepOne Plus real-time PCR Detection System (Applied Biosystem, Waltham, MA, USA). All target mRNAs levels were normalized to GAPDH, and miRNAs to miR24 as housekeeping genes. Relative quantification (RQ) for mRNA and miRNA were calculated using the ∆∆ cycle threshold (∆∆C*_T_*) method, with fold changes using the formula, as described in the section before. RQ for T1 or T2 were calculated using the formula 2^−ΔΔCt^ expressed relative to the mean values for the control group [43].

### 4.7. 16S rRNA Gene Amplification and Sequencing and Microbial Metabolites

For sequencing microbial composition, all fasting samples were analyzed by Biomes NGS GmbH (Wildau, Germany) via 16S rRNA gene amplification and sequencing. Microbial genomic DNA from fecal material was extracted by bead-beating technique. As the most promising for bacterial and archaeal primer pairs [44], the V3–V4 region of the 16S rRNA gene was amplified and sequencing was performed on the Illumina MiSeq platform using a 2 × 300 bp paired-end protocol, according to the manufacturer’s instructions (Illumina, San Diego, CA, USA).

Microbial metabolites were analyzed using mass spectrometry at the Department of Nutritional Sciences, University of Vienna. The detection technique was established based on the published method 2-NPH or 3-NPH derivatized fatty acids analysis utilizing LC-MS [45]. The metabolites were detected by liquid chromatography coupled to mass spectrometry (LC-MS). Therefore, Ultimate 3000 (Thermo Fischer Scientific, Waltham, MA, USA) and a micrOTOF-Q II (Bruker Daltonics, Bremen, Germany) with an Atlantis T3 3 μm column (2.1 × 150mm, Waters, Milford, MA, USA) were used, and kept at 40 °C. 

### 4.8. Bioinformatics and Statistical Analysis

Data are presented as mean ± standard deviation (SD). Data were analyzed using IBM SPSS Statistics for Windows Version 22.0 (IBM Corp., Armonk, NY, USA) and graph pad prism (Version 6). The paired *t*-test was used to compare the different time points for parametric values and the Wilcoxon test was used for nonparametric values. Statistical significance was defined by a *p*-value < 0.05.

Raw microbial sequences were processed using the Quantitative Insights Into Microbial Ecology (QIIME) pipeline [46]. High-quality reads were binned into operational taxonomic units (OTUs) at a 97% similarity threshold using UCLUST [47] and a “de novo” approach. Taxonomy was assigned using the Ribosomal Database Project (RDP) classifier against Greengenes database. All singleton OTUs were removed in an attempt to discard the majority of chimera sequences. Microbial alpha diversity was analyzed by using the Chao1 index, Shannon entropy, Simpson‘s index, and phylogenetic diversity whole tree metrics and beta diversity was estimated based on Bray–Curtis dissimilarity index and plotted as a multidimensional scaling or Principal Coordinates Analysis (PCoA) by the CLC Genomics Workbench version 20.0.4 (QIAGEN). The Mann–Whitney U test was used to analyze the mean difference of the alpha diversity index using GraphPad Prism version 9.0.0 (San Diego, CA, USA) and plotted as mean ± SD. *p*-value < 0.05 was considered statistically significant.

The difference in the microbial community composition (beta diversity) of the groups was tested using the permutational multivariate analysis of variance (PERMANOVA).

## 5. Conclusions

RD or fasting, in any version, beneficially changes clinical blood parameters, which has already been studied and documented. In addition, in animal models, RDs have been documented to increase lifespan by activation of SIRTs. We show that in humans, five days of consecutive nutrition depletion increases *SIRT1* and *SIRT3* expression in comparison with non-fasting controls. Additionally, PF leads to a switch in the gut microbial composition. Interestingly, the abundance of *Christensenella*, which is associated with longevity, increased after PF. To our knowledge, this is the first study assessing *SIRT* expression, including its interaction with the gut composition in PF subjects. 

## Figures and Tables

**Table 1:** Characterization of study population by intervention groups. Values for characterization are given in total numbers and as a percentage. Anthropometric measurements were given in mean ± SD.

**Characteristics**	**Total Study Population** ***n* (%)**	**Fasting Population** ***n* (%)**	**Non-Fasting Control** ***n* (%)**
Population size	51 (100)	20 (39.2)	31 (60.8)
Male	16 (31.4)	5 (25)	11 (35.5)
Female	35 (68.6)	15 (75)	20 (64.5)
BMI < 25 kg/m^2^ T1	27 (52.9)	6 (30)	21 (67.7)
BMI > 25 kg/m^2^ T1	18 (35.3)	9 (45)	9 (29)
BMI > 30 kg/m^2^ T1	6 (11.8)	5 (25)	1 (3.2)
**Characteristics**	**Total study population** **mean ± SD**	**Fasting population** **mean ± SD**	**Non-fasting control** **mean ± SD**
Age	45.24 ± 14.625	56.55 ± 12.576	37.94 ± 10.767
Weight kg at T1	75.882 ± 12.8509	80.550 ± −13.7093	72.871 ± 11.5029
BMI kg/m^2^ at T1	25.9298 ± −3.932	27.7610 ± 4.32706	24.7484 ± 3.20067
Weight loss kg T2–T1	−2.0067 ± 2.66021	−4.265 ± 1.77535	−0.2 ± 1.5

## Data Availability

No new data were created or analyzed in this study. Data sharing is not applicable to this article.
